# Contribution of nonconsensus base pairs within ArsR binding sequences toward ArsR-DNA binding and arsenic-mediated transcriptional induction

**DOI:** 10.1186/s13036-019-0181-4

**Published:** 2019-06-06

**Authors:** Xingjuan Chen, Xin Jiang, Cuijuan Tie, Jinnon Yoo, Yan Wang, Meiying Xu, Guoping Sun, Jun Guo, Xianqiang Li

**Affiliations:** 1 Guangdong Provincial Key Laboratory of Microbial Culture Collection and Application, Guangdong Institute of Microbiology, Guangzhou, China; 2 State Key Laboratory of Applied Microbiology Southern China, Guangzhou, China; 3 Science and Technology Library of Guangdong Province, Guangdong Institute of Science and Technology Information and Development Strategy, Guangzhou, China; 4 Signosis Inc., 1700 Wyatt Drive, suite10-12, Santa Clara, CA USA

**Keywords:** Bacterial biosensor, Arsenic bioreporter, Nonconsensus base pair, Arsenic repressor, Arsenic binding sequence, Protein-DNA recognition

## Abstract

**Background:**

A transcriptional reporter is the key component in bacterial biosensors which are employed to monitor the induction or repression of a reporter gene corresponding to environmental change. Interaction of a transcription factor with its consensus sequence generated by using a position weight matrix (PWM) model is crucial for its sensitivity of the reporter. However, recent studies suggest that PWM model based on independent contribution of individual consensus base pairs to protein interaction is often insufficient to explain complex regulation, such as the effect of nonconsensus sequences on the protein-DNA binding affinity. In the present study, we employed a simpler prokaryotic arsenic repressor (ArsR) regulation system to access the protein-DNA recognition. Contribution of nonconsensus base pairs within ArsR binding sequences toward ArsR-DNA binding and arsenic-mediated transcriptional induction was studied.

**Results:**

We constructed a series of arsenic responsive reporters, each comprising two copies of the ArsR binding sequences from different resources. We found that high arsenic-mediated induction specifically requires the binding sequence from *Escherichia coli* to be placed at the first binding sequence; however, no such preference was observed for the second binding sequence, which could be from *Acidithiobacillus ferrooxidans*, plasmid R773, *Synechococcus*, or a core binding sequence of *ars*R. By creating a series of reporters differed at the nonconsensus base pairs of the second binding sequence, we observed that some constructs bound weakly while others strongly to ArsR. Most interestingly, although a number of these reporters showed similar binding affinity to ArsR, their arsenic-dependent induction differed significantly.

**Conclusions:**

The results indicated that nonconsensus base pairs could have profound influence on protein binding and may also modulate post-binding function. These findings provide new insights into the complex regulation of gene expression and facilitate the development of transcriptional reporter-based biosensors.

## Background

The interaction between a transcription factor (TF) and its corresponding DNA binding sequence is crucial in gene regulation [1, 2]. The base composition determines the binding affinity of the sequence to the TF. The contribution of individual base pairs in the interaction with a TF can be assessed by their conservation. Algorithms typically use the statistically simple position weight matrix (PWM) model for a binding consensus sequence [3, 4]. Moreover, the binding consensus sequence can be determined by sequencing a group of DNA fragments or oligonucleotides selected by a TF using in vitro methods such as ChIP-seq or SELEX [5]. Nevertheless, more than 40% of TFs still remain unknown for their binding sequences [5]. In *Escherichia coli* (*E. coli*), most TFs bind to a single binding site in chromosomal DNA, such as arsenic repressor (ArsR), a metalloregulatory transcriptional repressor to its operator/promoter (O/P) sequence [6]. Due to the abundant presence of ArsR binding sequences in microbial chromosomes, the alignment of these binding sequences via comparison and analysis with PWM can lead to the identification of its binding consensus sequence or motif [7]. However, recent studies suggest that PWM model based on independent contribution of individual consensus base pairs to protein interaction is often insufficient to explain various complex regulations [8], such as the effect of nonconsensus sequences on the protein-DNA binding affinity. In the present study, we employed a simpler prokaryotic ArsR regulation system to access the protein-DNA recognition.

ArsR, belonging to the Smt/ArsR family, is a regulatory protein that controls the expression of the genes involved in arsenical resistance via interaction with the arsenic-responsive operon. ArsR binding prevents the RNA polymerase from interacting with the O/P sequence of its targeted genes in the absence of arsenicals [7, 9]. Upon arsenic binding, the protein dissociates from the promoter, subsequently activating the gene expression [9–11]. ArsR protein is well characterized in plasmid R773 and *E. coli* chromosome. Both of these ArsR proteins are able to form homodimer, each with a Cys32-Val-Cys-Asp-Leu-Cys arsenic-binding sequence located at the start of their DNA binding domain [11]. ArsR from *Acidithiobacillus ferrooxidans* (*A. ferrooxidans*) does not have the binding sequence at this location, instead, their cysteine residues are located at amino acid residues of 95, 96, and 102 [12]. Both binding and the consensus sequences of Smt/ArsR family proteins, including those in *A. ferrooxidans*, have been characterized [6, 7, 12–14].

In a previous study, we created two arsenic reporters, pLHPars9 and pLLPars9, in order to rapidly and cost-effectively monitor arsenic on site and measure arsenic bioavailability. The bioreporters pLHPars9 and pLLPars9 comprised either a high or low copy-number plasmid, along with common elements of ArsR-luciferase fusion and addition of two binding sequences, one each from *E. coli* (ECBS) and *A. ferrooxidans* (AFBS) chromosome, before the R773 *arsR* operon (*arsRBC*) [15]. Both of these reporters were highly sensitive to arsenite, with a low detection limit of 0.04 μM arsenite (~ 5 μg/L) and differed in their metal specificity, with pLLPars9 being more specific to arsenite and pLHPars9 to both arsenite and antimonite. The only difference between pLHPars9 and pLLPars9 is their copy numbers.

In the present study, we constructed a set of arsenic bioreporters comprising two copies of different binding sequences. We found that high arsenic-mediated induction specifically requires ECBS to be placed at the first binding sequence; however, no such preference was observed for the second binding sequence. By creating a series of reporters differed at the nonconsensus base pairs of the second binding sequence, we tested the interaction of these probes with the protein. Interestingly, while some of the nonconsensus base pairs resembling the consensus are needed for the interaction with the ArsR protein, some of the nonconsensus base pairs appear to also affect the post-binding function of the TF.

## Results

### Arsenic transcriptional induction with a promoter containing ECBS binding sequence in arsenic bioreporters

**Fig. 1:**
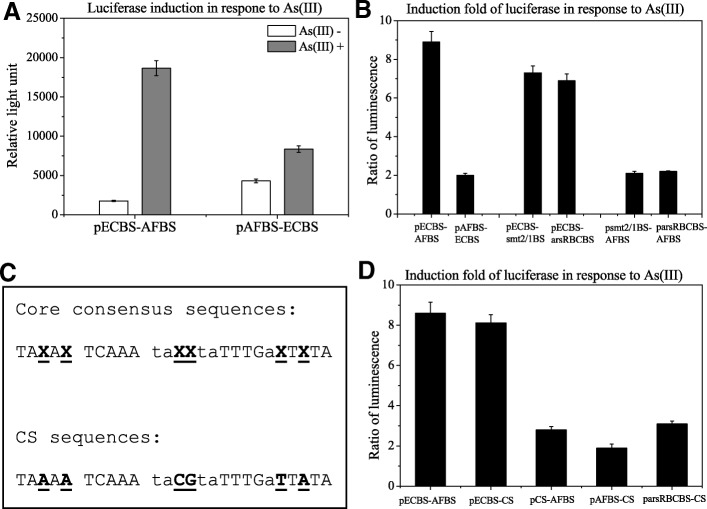
Luciferase analysis of reporter constructs containing different combinations of two binding sequences. **a**: Luciferase activities of pECBS-AFBS and pAFBS-ECBS were measured and compared between the lysates prepared from cells treated with (grey) and without (open) 10 μM arsenite for 1 h. **b**: Luciferase activities ratios of untreated to arsenic-treated cells were determined. The ratios were compared between pECBS-AFBS and pAFBS-ECBS, pECBS-smt2/1BS and pECBS-arsRBCBS, and psmt2/1BS-AFBS and parsRBSBS-AFBS. C: Sequences of ArsR binding core motif and CS, assigned nucleotides marked with underline. D: Luciferase activities ratios of untreated to arsenic-treated cells were determined. The ratios were compared among pECBS-AFBS, pECBS-CS, pCS-AFBS, pAFBS-CS and parsRBCBS-CS

In a previous study, we found that a luciferase reporter construct pLLPars9 (or pECBS-AFBS in this study) containing ECBS-AFBS comprising two copies of ArsR binding sequences (BS), one from *E. coli* chromosome (EC) and another from *A. ferrooxidans* (AF) chromosomal DNA, responded better more robustly to arsenic treatment than the reporters comprising either one or two identical copies of EC or AF [15]. In this study, we swapped the position of ECBS and AFBS to create pAFBS-ECBS. After transformed into DH5a, luciferase activates of pECBS-AFBS and pAFBS-ECBS were measured and compared (Fig. 1a). Relative to untreated control cells, pAFBS-ECBS showed only 2-fold induction in arsenic-treated cells, compared to the 9-fold induction with pECBS-AFBS (Fig. 1b). This dramatic induction difference suggested that the order of these two binding sequences is crucial in the arsenic-mediated induction of the reporter.

Furthermore, we replaced the AFBS moiety within ECBS-AFBS with the binding sequence of *Synechococcus* smt2/1 (smt2/1BS) or arsRBC (arsRBCBS), to create the reporters pECBS-smt2/1BS and pECBS-arsRBCBS, and compared the luciferase activities of cell lysates prepared from their transformed cells with or without arsenic treatment. As presented in Fig. 1b, the induction of ECBS-smt2/1BS and ECBS-arsRBCBS moderately declined, losing approximately 15–25% induction folds of ECBS-AFBS. This suggested that AFBS at this position is not crucial for induction and can be substituted by other ArsR binding sequences. When we replaced ECBS moiety within ECBS-AFBS with the binding sequence of smt2/1 or arsRBC to create reporters psmt2/1BS-ECBS and parsRBCBS-ECBS, we found that the ratio of luciferase activities significantly declined, losing approximately 70% compared to ECBS-AFBS, as shown in Fig. 1b. The aforementioned results demonstrated that ECBS needs to be the first binding sequence in order to robustly respond to arsenic.

The consensus sequence of a DNA-binding protein can be determined by comparison of a group of binding sequences. Those consensus base pairs are believed to be crucial for the protein to bind DNA and the nonconsensus base pairs are not important to the binding. Arsenic binding proteins from different microbes are DNA-binding proteins. The DNA sequences that they bind to display a consensus sequence [7]. Our above data indicated that the second binding sequence within the biosensors was relative flexible, which could be more tolerant to bioengineering manipulation, such as a consensus sequence. According to the consensus sequence of arsRBC and cadCA, we designed a binding sequence CS (Fig. 1c) and swapped it with the AFBS moiety to construct pECBS-CS, with 3 Ts in between. Luciferase assay revealed that pECBS-CS showed no significant difference in the response to arsenic treatment when compared to pECBS-AFBS, suggesting that the CS can be used to replace AFBS within the biosensors. However, when we swapped ECBS with the CS to make pCS-AFBS, it demonstrated a significant change (Fig. 1d). Moreover, when we replaced ECBS of ECBS-CS with arsRBCBS or AFBS to construct parsRBCBS-CS and pAFBS-CS, they lost induction significantly like any other constructs without ECBS being at the first position as shown as above. These results with CS indicated that ECBS must be the first binding sequence.

### Arsenic cannot remove the repressor protein from AFBS-ECBS and CS-ECBS binding sequences

**Fig. 2:**
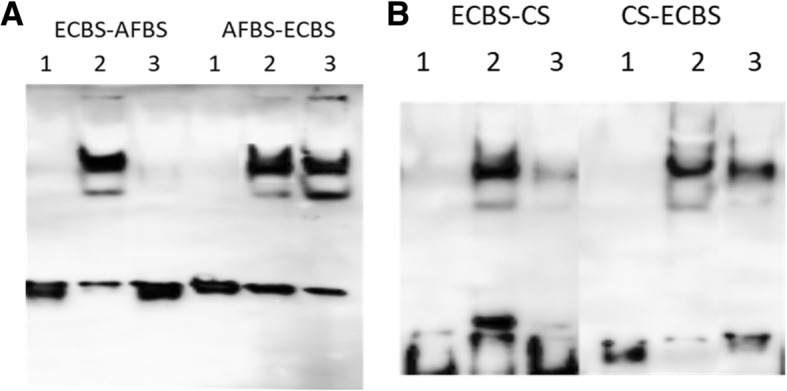
Comparison of biotin-labeled probes in protein binding with EMSA. Biotin-labeled probes (1) were mixed with lysates prepared from untreated control cells (2) or arsenic treated cells (3) respectively and subjected to EMSA. **a**: Probe ECBS-AFBS was compared with AFBS-ECBS. **b**: Probe ECBS-CS was compared with CS-ECBS

To examine whether there exists any difference between ECBS-AFBS and AFBS-ECBS in ArsR binding, we performed EMSA. Biotin-labeled probes of ECBS-AFBS and AFBS-ECBS were mixed with lysates prepared from cells with and without arsenic treatment, respectively. As previous reported, two shifted bands were observed with the ECBS-AFBS probe in mock-treated cell lysate [15] and the intensity of both shifted bands dramatically declined in arsenic-treated cells (Fig. 2a), indicating that the arsenic treatment disrupted the interaction between the probe and the bound repressor protein. In contrast, when we utilized the AFBS-ECBS probe to execute EMSA, we found no difference in both the number and intensity of the shifted bands between mock-treated and arsenic-treated cells. This indicated that the arsenic treatment was unable to remove the repressor protein from the AFBS-ECBS (Fig. 2a).

Next, we compared ECBS-CS and CS-ECBS probes with EMSA. The ECBS-CS probe revealed two shifted bands in control cell lysate and much weaker intensity of the shifted bands in arsenic-treated cell lysate (Fig. 2b). Again, we observed no significant difference in the intensity of the shifted bands between control and treated cells with CS-ECBS probe. This result is in accordance with AFBS-ECBS, suggesting no removal of the repressor protein from CS-ECBS probe under arsenic treatment.

### Arsenic removal of the repressor protein from ECBS-CS binding sequence required a linker of 3Ts

**Fig. 3:**
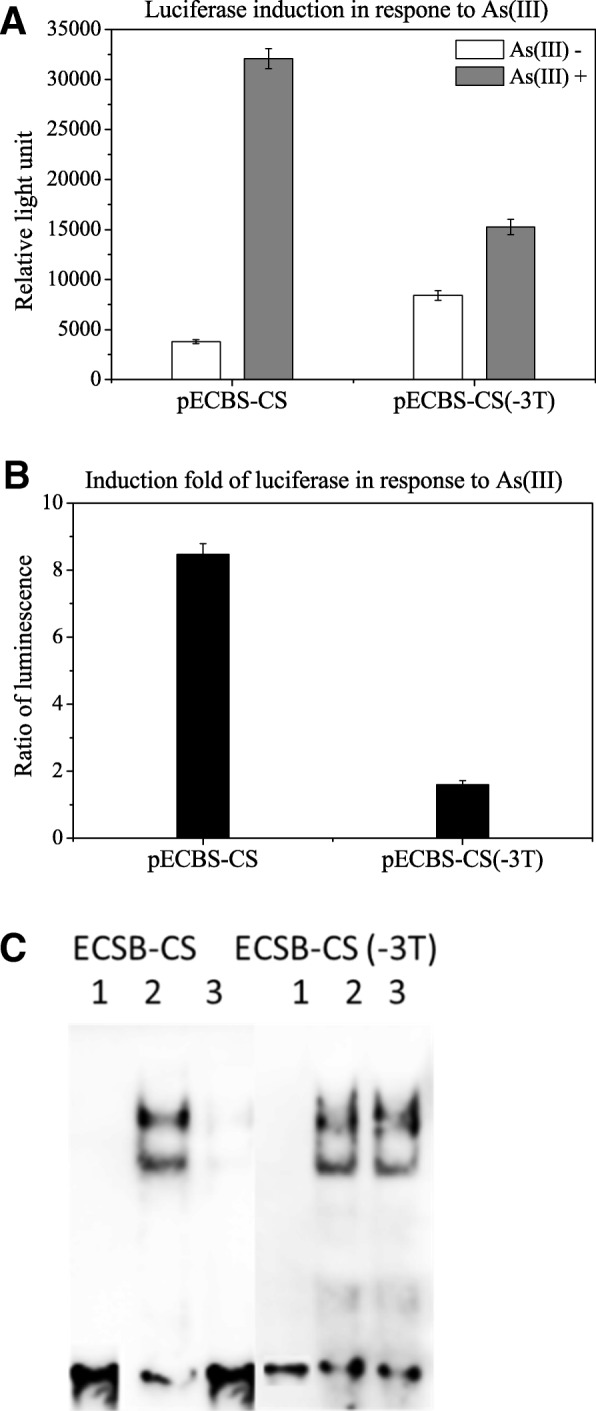
Analysis of ECBS-CS and ECBS-CS(− 3 T) using luciferase and EMSA. A: Luciferase activities ratios of untreated to arsenic-treated cells were determined. B: Probes of ECBS-CS and ECBS-CS(− 3 T) were mixed with lysates prepared from untreated and arsenic-treated cells and subjected to EMSA

As a linker of 3Ts was inserted between ECBS and CS, we examined whether it was necessary for the induction by removing the linker from pECBS-CS to create pECBS-CS(− 3 T). After transformation, the luciferase activity of pECBS-CS(− 3 T) transformed cells were treated with and without arsenite. A significant reduction of luciferase activity in pECBS-CS transformed cells with arsenic treatment was not observed when compared to untreated control cells; however, it was observed in the pECBS-CS(− 3 T) cells treated with arsenic (Fig. 3a; b). This result suggested that the linker of 3Ts is needed for arsenic-mediated induction.

Moreover, to examine whether the absence of 3Ts caused a steric hindrance for binding of the dimers to the binding sequences or prevented the removal of the bound repressor protein from the bound sequence, we performed EMSA with biotin-labeled probes of ECBS-CS and ECBS-EC(− 3Ts). As shown in Fig. 3c, like ECBS-AFBS probe, the result with ECBS-CS probe displayed two shift bands in arsenic-untreated cells but significant decline in arsenic-treated cells. Without the linker, ECBS-CS(− 3 T) displayed two shifted bands in both control and treated cells, indicating the absence of any interference with protein binding, thus ruling out the possibility of steric hindrance. Therefore, this result suggested that the absence of the linker hampered arsenic-mediated removal of the repressor protein from the bound sequence.

### Fast analysis of the DNA binding sequences of ArsR with DNA filter assay

The above results indicated that two ArsR binding elements within the biosensors were needed in order to have a sensitive response to arsenic treatment. The first element must be from *E. coli* and the second one was more flexible, such as arsRBCBS or CS. Although arsRBC of pECBS-arsRBCBS and CS of pECBS-CS contained the same consensus sequence, their responses to arsenic treatment were distinct with moderate difference. We assumed that the difference could arise only from the contribution of nonconsensus base pairs of the second binding sequence. To investigate the contribution of the nonconsensus base pairs in both binding and induction, we constructed a series of probes and reporters exclusively with alternative nonconsensus base pairs in the second binding site within ECBS-arsRBCBS. According to the consensus sequence of arsRBC, only 4 base pairs are not conserved. Investigation of different combinations of these 4 base pairs required testing of a large series of probes.

**Fig. 4:**
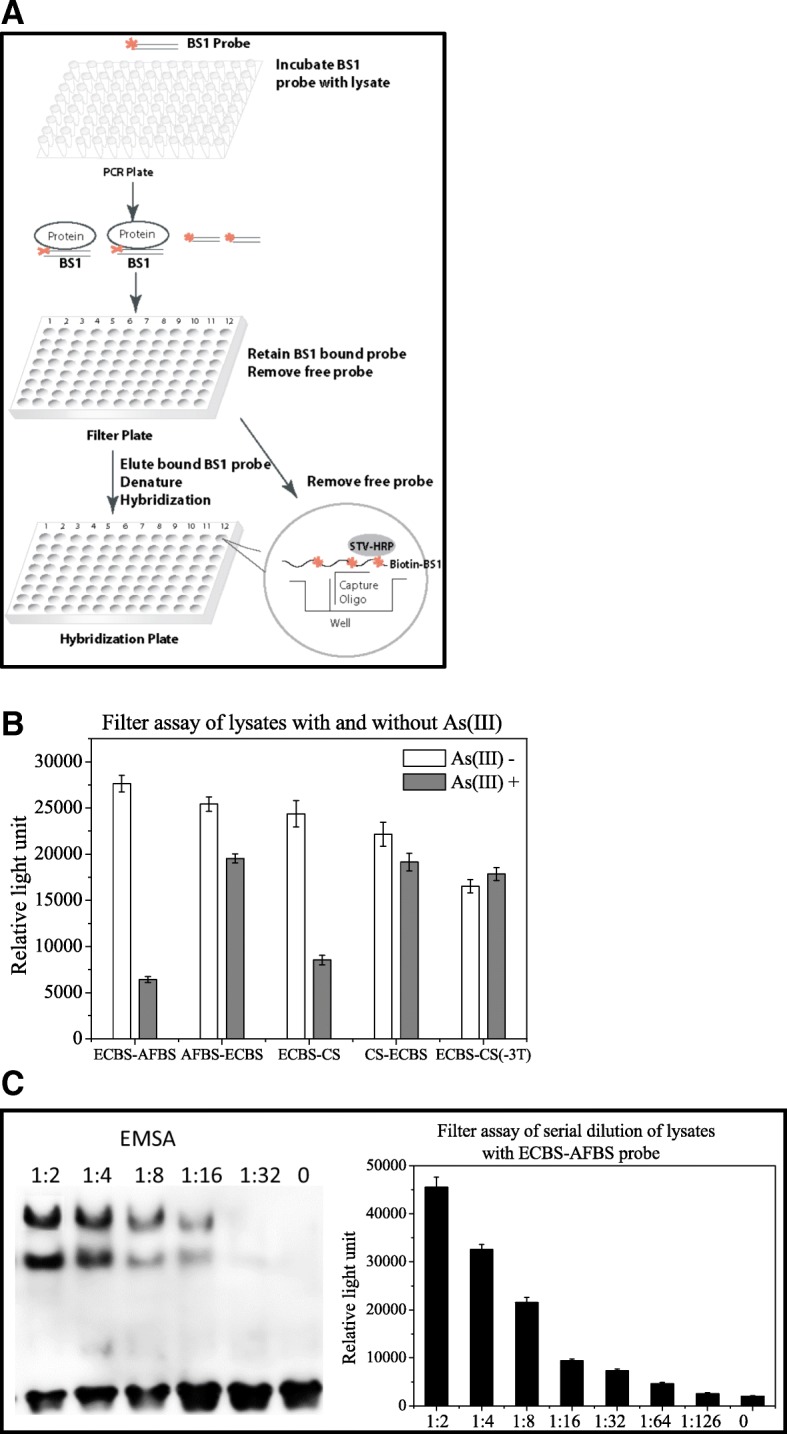
Filter assay. **a**: Schematic diagram of the filter assay comprising 3 steps; the biotin-labeled (* indicates biotin labeled) probe BS1 (Binding Sequence) was first mixed with lysates; BS1 probe bound to the protein in the cell lysate to form protein/DNA complexes; second, the mixture loaded onto nitrocellulose (NC) membrane-based filter plate, the protein/DNA complexes retained on membrane, the free probe washed away, and the protein-bound probe eluted, denatured and hybridized on the hybridization plate; third, the hybridization and detection within one of 96 wells was illustrated in the Cycle. The capture oligos were pre-coated on the bottom of the well, the denatured biotin-labeled probe BS1 hybridized to the capture oligo, and further detected with Streptavidin *Horseradish Peroxidase (STV-HRP) and measured with a luminescence plate reader*. The *luminescence* signal directly corresponds to the binding activity. **b**: Filter assay of probes ECBS-AFBS, AFBS-ECBS, ECBS-CS, CS-ECBS and ECBS-CS(− 3 T) using lysates prepared from cells treated with (grey) and without (open) 10 μM arsenite for 1 h. C: Serial 2-fold dilutions of *E. coli* DH5α cell lysates were mixed with ECBS-AFBS probe. The mixtures were analyzed with EMSA and the filter assay respectively. The filter assay data shown are the mean values (±standard deviation) obtained from three independent experiments

EMSA is usually used for monitoring protein/DNA interactions. Due to the low throughput nature of the assay, the analysis becomes time consuming when handling a large sample size. We therefore developed a fast filter binding assay that enabled us to efficiently monitor the interaction of several probes with their binding proteins simultaneously (Fig. 4a). In this assay, probes were first mixed with lysates prepared from *E. coli* treated with or without arsenite, respectively. After incubation, the mixtures were loaded onto a nitrocellulose membrane (NC)-coated 96-well plate. Only protein-bound probes could stay on the plate and free probes passed through the membrane upon centrifugation. After washing, the plate was then treated with sodium dodecyl sulfate (SDS) to denature proteins, thereby releasing the probes. The released biotin-labeled probes were subjected to hybridization using a plate pre-coated with complementary sequences, and further monitored with streptavidin-HRP for luminescent detection.

To examine the feasibility of the filter assay, we employed probes ECBS-AFBS, AFBS-ECBS, ECBS-CS, CS-ECBS and ECBS-CS(− 3 T) and mixed them with lysates prepared from arsenic-treated and mock-treated cells. The probe mixtures with lysates were first validated with EMSA before using for the filter assay. The filter assay indicated that the binding of ECBS-AFBS probe with the lysate without arsenic treatment was much stronger than the lysate with arsenic treatment, and the ratio of the binding intensities of control to treated cells was about 5-fold (Fig. 4b). As expected, binding of the AFBS-ECBS probe with control and arsenic treated cell lysates was both strong and no obvious difference in binding was observed. The result with ECBS-CS probe was similar to that with the ECBS-AFBS probe and the binding ratio of control to treated cells was about 3-fold. In addition, both ECBS-CS(− 3 T) and CS-ECBS probes displayed no difference in the binding ratio of the two different lysates (Fig. 4b). These results demonstrated that the filter assay in general was in accordance with EMSA. The only difference of the filter assay was that it could not present two distinct shifted bands like EMSA.

Next, we employed both assays to perform two-fold dilutions of lysates from both arsenic treated and control cells with the ECBS-AFBS probe. As shown in Fig. 4c, EMSA could detect the complex in 1:16 diluted lysates and filter assay in 1:64, indicating that the filter assay is 4 times more sensitive than EMSA. Therefore, the filter binding assay was able effectively to analyze the binding of several probes with target protein in a quick mode.

### Identification of alternations at nonconsensus base pairs crucial in protein binding using DNA filter assay

The original *E. coli* ArsR binding sequence was identified as acacattcg TT AA GT CA TA TA (TG) TT TT TG AC TT A [6]. Based on the comparison with other ArsR binding sequences, we noted an extra tail of 9 base pairs at the 5′ end that are unlikely to contribute to arsenic-mediated induction. To reduce the cost in oligonucleotide synthesis, we removed 5 base pairs at the 5′ end to make sECBS as ttcg TT AA GT CA TA TA (TG) TT TT TG AC TT A. Functional analysis using reporters with the shorter version, sECBS to replace ECBS revealed no difference (data not shown).

**Fig. 5:**
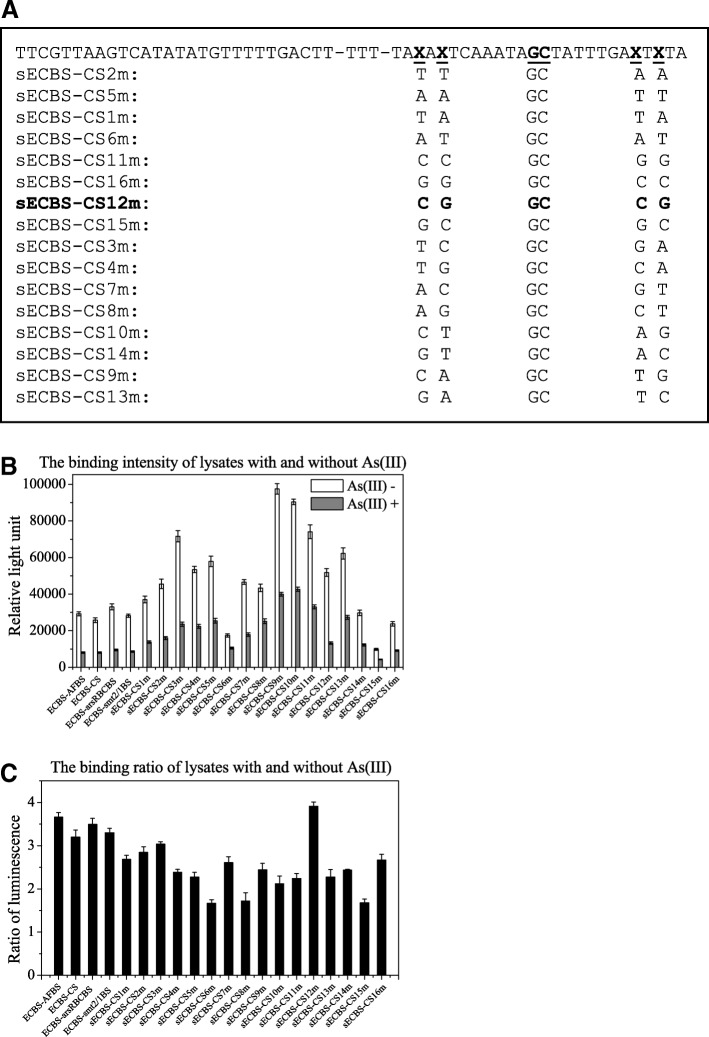
Filter assay and luciferase analysis of probes with alterations at the nonconsensus base pairs. **a**: List of the probes with alternations at nonconsensus base pairs. **b**: The binding intensities of the probes were measured with filter assay using lysates prepared from cells treated with (grey) and without (open) 10 μM arsenite for 1 h. **c**: Luciferase activities ratios of untreated to treated lysates with these probes were determined. Data shown are the mean values (±standard deviation) obtained from three independent experiments

Among the 4 base pairs that are not conserved, two base pairs locate on each side of the inverted repeat region, TAxAxTCAAATA xx TATTTGAxTxTA, the core binding sequence was obtained by the alignment of ArsR binding sequences among O/P sequences of arsRBC, cadCA, smtS2/S1, smtS4/S3, ziaA, czrAB, and nmtA [7]. To investigate the contribution of these nonconsensus base pairs to ArsR binding, we systematically designated different nucleotides at the position on the left and created complementary nucleotides on the right of the repeat. We constructed a series of probes with AA, TT, TA, AT CC, GG CG, GC, TC, TG, AC, AG, CT, GT, CA, and GA on the left side of the repeat, as presented in Fig. 5a. Biotin-labeled probes were employed to execute the filter binding assay using cell lysate of *E. coli,* with or without arsenic treatment. As shown in Fig. 5b, certain probes such as sECBS-CS9m and sECBS-CS10m bound to the protein from both cell lysates, with or without arsenic treatment, were stronger than ECBS-AFBS, sECBS-arsRBCBS, or sECBS-CS, whereas another probe sECBS-CS15m bound to the protein from both cell lysates, with or without arsenic treatment, was weaker than ECBS-AFBS, sECBS-arsRBCBS, or sECBS-CS. Therefore, the nonconsensus base pairs could change the binding affinity to both directions. In addition to the alternations at nonconsensus base pairs, we included an alternation in the consensus base pairs to construct the probes ECBS-CS-SM as a control. With the filer binding assay, we observed that a change of 2 base pairs in the consensus sequence destroyed the difference in binding between cell lysates with and without arsenic treatment (data not shown). Moreover, we found one probe sECBS-CS12m bound the protein strongly in control cells, but weakly in arsenic-treated cells. The binding difference of this probe in control versus treated cells appeared to be the biggest among all other probes (Fig. 5c).

### Contribution of nonconsensus base pairs to protein binding and luciferase induction

**Fig. 6:**
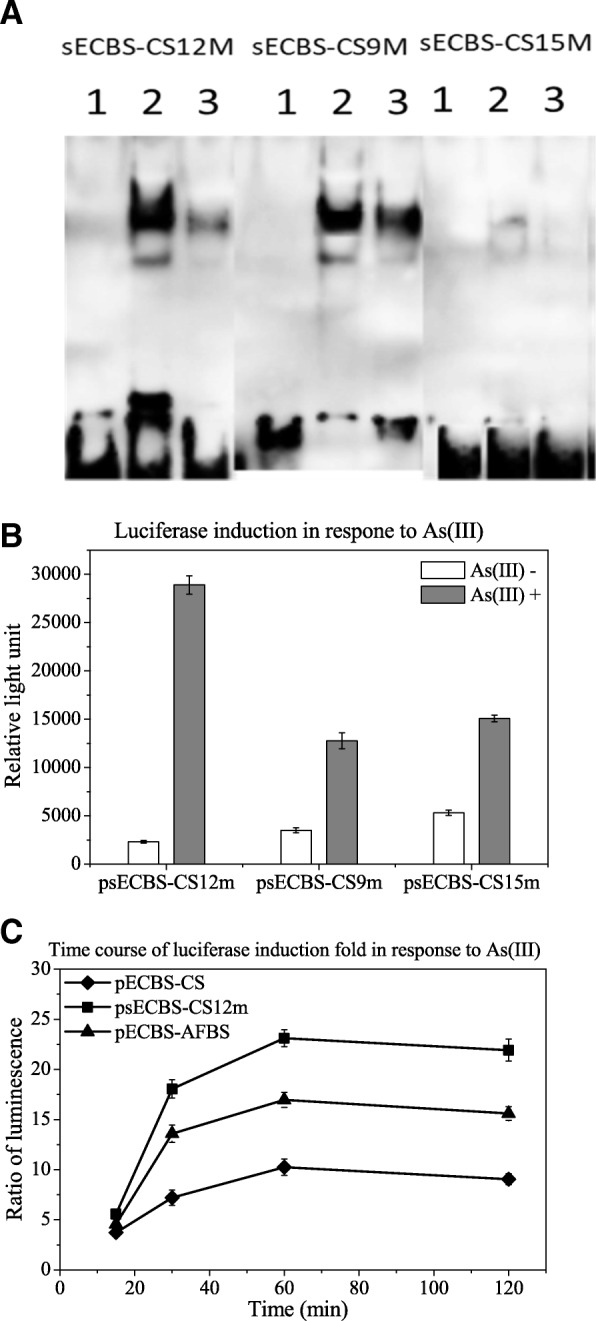
EMSA and luciferase analysis of reporter constructs with the binding sequences. **a**: Biotin-labeled probes of sECBS-CS9m, sECBS-CS12m and sECBS-CS15m were mixed with lysates prepared from untreated (open) and arsenic-treated cells (grey) and subjected to EMSA. **b**: Luciferase activities of sECBS-CS9m, sECBS-CS12m and sECBS-CS15m transformed cells pre-treated with or without 10 μM arsenite for 1 h were measured. **c**: Time course of luciferase induction of pECBS-CS12m (square) transformed cells pre-treated with 10 μM arsenite for 15, 30, 60 and 120 min were determined and compared with pECBS-AFBS (triangle) and pECBS-CS (diamond). Data shown are the mean values (±standard deviation) obtained from three independent experiments

From the filter binding assay, we chose three probes, the strongest sECBS-CS9m in binding, the weakest sECBS-CS15m in binding, and the highest sECBS-CS12m in induction, to validate the results with EMSA. As shown in Fig. 6a, the intensities of the shifted bands in EMSA were equivalent to the binding strengths in the filter binding assay, sECBS-CS9m being the strongest and sECBS-CS15m being the weakest. The difference of sECBS-CS12m shifted bands in binding intensity is the highest between control and induced cells. These results again demonstrated that changes in nonconsensus base pairs could lead to critical differences in protein binding. Importantly, our data also revealed that changes in nonconsensus base pairs could enhance arsenic-mediated removal of the bound protein, an additional functional impact after the binding is established.

To investigate the induction of few binding sequences, we used sECBS-CS12m, sECBS-CS9m, and sECBS-CS15m to replace the ECBS-AFBS of pECBS-AFBS, in order to construct psECBS-CS12m, psECBS-CS9m, and psECBS-CS15m. As expected, psECBS-CS12m resulted in a better induction than the other two reporters psECBS-CS9m and psECBS-CS15m (Fig. 6b). Furthermore, we compared psECBS-CS12m with pECBS-AFBS and pECBS-CS. The transformants were treated with 10 μM arsenite for 15, 30, 60 and 120 min. As shown in Fig. 6c, arsenic-mediated induction of psECBS-CS12m was significantly better than that with either pECBS-AFBS or pECBS-CS.

## Discussion

Arsenic, as a naturally occurring element, is widely distributed throughout the environment. Long-term exposure to arsenic from drinking water and food can cause human diseases [16]. Prevention of further exposure to arsenic needs rapid and cost-effective on-site analytical techniques to monitor arsenic in water supplies. Bacteria-based assays are an emerging technology, in the case of arsenical contamination, to monitor arsenic-induced gene expression. Compared to the traditional capital equipment-based methods that are inappropriate for on-site detection, bacteria-based assays are robust and inexpensive for detecting arsenic in the field [17]. More significantly, they could measure arsenic bioavailability that accounts for the difference between exposure and dose [18]. The crucial component of bacteria-based assays is the reporter, comprising a promoter/operator (or an operon) and a reporter gene [19]. Ideally, a good reporter should display high sensitivity and specificity, low endogenous background, and a wide dynamic range of response [20]. In our previous study of making sensitive arsenic reporter, we constructed pLLPars9 (the same construct as pECBS-AFBS in this study) reporter and demonstrated that it is equivalent to some of the best reporters constructed to date in response to arsenic [15]. In this study, we demonstrated that the reporter psECBS-CS12m is significantly better than pLLPars9.

Metal-inducible operons contain an imperfect 12–2-12 inverted repeat, except the smt operon having two inverted repeats S2/S1 and S4/S3. Each repeat is occupied by an ArsR homodimer. In the previous study, we designed, such as smt operon, two binding sequences ECBS-AFBS and found that the induction of the luciferase reporter in response to the treatment of arsenic is better than either of single binding sequences [15]. We also found that the induction with these two different binding sequences is better than the two identical sequences either from EC or AF. In the present study, we uncovered that ECBS must be at the front position and the induction dramatically declined if it was replaced by other binding sequences such as smt2/1 or arsRBC. In contrast, AFBS at the second position can be replaced by other binding sequences without affecting the induction to a significant degree. This indicated that an appropriate order of these two binding sequences is important for achieving maximal induction. As the two binding sequences bind to two dimers, the complex could be stabilized by dimer-dimer interaction [7]. Change in the order of these two binding sequences, that is AFBS-ECBS, could allow the binding of two dimers, but the order might affect arsenic interaction with the repressor protein or removal of the repressor protein from the binding sequences. Therefore, unlike ECBS-AFBS, arsenic binding sites within AFBS-ECBS might be hidden due to steric structure, which prevents arsenic binding or dissociation the repressor from the binding sequence.

Protein-DNA recognition has been increasingly appreciated to be more complex than previously thought. Although the simple model of PWM has been widely used to define the DNA binding motifs of individual TFs, recent studies suggest that this model based on independent contribution of individual consensus base pairs to protein interaction is often insufficient to explain various complex regulation [8], such as the relevant dinucleotides or trinucleotides crucial to protein-DNA recognition [21–24], significant difference of low-affinity binding sites from the consensus sequence [25, 26], novel DNA-binding specificities of multi-protein complexes formed with a TF [27–29], and the effect of flanking sequences on the binding affinity [30]. In the present study, we employed a simpler prokaryotic ArsR regulation system to access the protein-DNA recognition. We found base pairs at nonconsensus positions within the second binding sequences such as ECBS-CS15m could result in lower binding with target protein, whereas others such as ECBS-CS9m resulted in binding with higher affinities, although both still maintained the consensus sequence. PWM was unable to explain these results. More interestingly, our study demonstrated that one of the base pairs at the nonconsensus position could also affect induction, the function beyond DNA binding. We found sECBS-CS12m, which could bind to target protein as well as ECBS-CS; however, its response to arsenic was much stronger than ECBS-CS. Their similar basal binding levels but differential induction rates suggest that arsenic-mediated removal of the binding protein from the DNA binding sequence of CS12m is faster than CS. Therefore, like AFBS-ECBS, the interaction of these nonconsensus base pairs with the repressor protein could influence arsenic binding or arsenic-induced conformational change of the repressor protein, leading to differential turnover of the bound protein from the binding sequence, as exemplified by the observations that AFBS-ECBS was no longer sensitive to arsenic while sECBS-CS12m became more sensitive to the arsenic.

## Conclusions

In the present study, we found that nonconsensus base pairs played important roles in protein-DNA binding and gene transcriptional regulation. More sensitive and accurate biosensors for arsenic detection can be developed through the design of nonconsensus base pairs. Our current findings illustrate an innovative strategy to construct better reporters, which will facilitate the development of more sensitive biosensors to monitor environmental arsenic via the induction of reporter gene expression.

## Methods

### Plasmid construction

Reporter constructs with different orders and sources of binding sequences were made by modifying the binding sequence of pLLPars9 [15], which was renamed as pECBS-AFBS in this study. The sense and antisense strand sequences were synthesized and annealed to generate double strand fragments with the sticky end of XbaI and HindIII, which were subsequently cloned into the XbaI and HindIII site of pLLPars9 [15] to replace the ECBS-AFBS to make constructs, pAFBS-ECBS, pECBS-smt2/1BS, pECBS-arsRBCBS, psmt2/1BS-ECBS, parsRBCBS-ECBS, pCS-AFBS, parsRBCBS-CS, pAFBS-CS, pECBS-CS, and pECBS-CS(− 3 T). Five base pairs at the 5′ end of ECBS were removed to make sECBS. Different nucleotides at the nonconsensus position of CS were designated to make CS1-16 m. Then sECBS and CS1-16 m were subsequently cloned into the XbaI and HindIII site of pLLPars9 [15] to replace the ECBS-AFBS to make constructs, sECBS-CS9m, sECBS-CS12m and sECBS-CS15m.

### Luciferase assay

*E. coli* DH5α competent cells were transformed with the recombinant plasmids constructed in this study. Single colonies were picked and inoculated in 2 mL Luria-Bertani (LB) media supplied with 25 μg/mL chloramphenicol for 12–16 h at 37 °C with vigorous shaking. The overnight culture was 1:50 diluted in a 1.5 mL microcentrifuge tube with pre-warm and fresh-prepared 2 mL LB media supplied with chloramphenicol. The diluted cells were cultured for additional 3 h at 37 °C until the optical density (OD) reached 0.5. Cells were treated with or without 10 μM sodium arsenite [As (III)] for 60 min at 37 °C. The cell samples were sonicated to lyse the cells, and the protein concentration was measured with Bradford Protein Assay (Bio-Rad, Cat#5000201) to confirm the equal protein concentration among the treated and untreated cell samples. Twenty μL of induced sample was taken and mixed 50 μL luciferase substrate, and the luciferase activities were measured on the luminescence plate reader (Veritas).

### Preparation of cell lysates

One mL of cell culture with or without sodium arsenite was centrifuged at 10,000 g for 1 min and the pellet was resuspended in 300 μL of lysis buffer (10 mM Tris-HCl, pH 8.0, 0.1 M NaCl, 1 mM ethylene diamine tetraacetic acid (EDTA), and 0.1% [w/v] polyethylene glycol octylphenol ether (TRITON X-100)). Senve point five μL of a freshly prepared lysozyme solution (10 mg/mL in 10 mM Tris-HCl, pH 8.0, final concentration is 0.25 mg/mL) was added and mixed well by tapping the tube gently, and the lysis mixture was incubated for 10–20 min at room temperature. After centrifugation, the supernatant was used for electrophoretic mobility shift assay (EMSA) or Filter assay.

### EMSA

One to 3 μg cell lysate was mixed with 2 μL of 5× binding buffer and 1 μL polyd(I-C) and incubated on ice for 5 min. One μL of biotin-labeled probe was added to the mixture and incubated at 22 °C for 30 min. Each reaction mixture was separated using a 6.5% non-denaturing polyacrylamide gel at 100 V at 4 °C in 0.5 × Tris-borate-EDTA (TBE) for about 50 to 60 min. After the gel was transferred onto an NC membrane and blocked by adding 15 mL of blocking buffer for 20 min at room temperature, the biotin-labeled probe on the blot was then detected with streptavidin–HRP and chemiluminescent substrates (enhanced chemiluminescence by luminol, Pierce). The image was acquired using an imager.

### Filter assay method

In this assay, 2 μL cell lysate (2–10 μg) was mixed with 10 μL 2× Binding Buffer Mix (40 mM 4-(2-hydroxyethyl)-1-piperazineethanesulfonic acid (HEPES), pH 7.6, 20 mM ammonia sulfate, 2 mM dithiothreitol (DTT), 20 mM KCl, and 0.4% Tween-20), 1 μL biotin-labeled probe, and 7 μL ddH_2_O in a 96-well PCR plate. After incubation at room temperature for 30 min, the reaction mix was loaded onto to a prewashed filter assay plate and incubated on ice for 20 min, following which it was centrifuged at 600 g for 2 min. The flow-through was discarded and the plate was washed for 4 times with filter wash buffer (100 mM Tris-HCl, pH 7.6, 2.5 mM EDTA, and 0.1% Tween-20). The bound probe was eluted with Elution buffer (0.5% SDS). The eluted probe was heated at 95 °C for 3 min before hybridization. Hybridization was carried out by adding the eluted DNA probe to a plate pre-coated with corresponding DNA and incubating at 42 °C overnight. After wash, the bound probe was eluted from the filter and collected for quantitative analysis through DNA plate hybridization. The captured DNA probe was further detected with streptavidin-HRP and the signals were read by a luminescence plate reader (Beckman Coulter, LD-400), and reported as relative light units (RLUs). Induction fold was the ratio of luminescence of arsenic-treated cells to that of arsenic-untreated cells.

## Data Availability

All data generated or analysed during this study are included in this published article.

## Abbreviations

AFBS: binding sequences from *A. ferrooxidans*
ArsR: arsenic repressor
arsRBC: R773 *ars*R operon
arsRBCBS: binding sequence from arsRBC
CS: consensus sequence of arsRBC and cadCA
DTT: dithiothreitol
ECBS: binding sequences from *E. coli*
EDTA: ethylene diamine tetraacetic acid
EMSA: electrophoretic mobility shift assay
HEPES: 4-(2-hydroxyethyl)-1-piperazineethanesulfonic acid
LB: Luria-Bertani media
NC: nitrocellulose membrane
O/P: operator/promoter
OD: optical density
PWM: position weight matrix
RLUs: relative light units
SDS: sodium dodecyl sulfate
smt2/1BS: binding sequence from *Synechococcus* smt2/1
TBE: Tris-borate-EDTA
TF: transcription factor
TRITON X-100: polyethylene glycol octylphenol ether
